# Classification and surgical treatment of the angled thumb

**DOI:** 10.1186/1753-6561-9-S3-A9

**Published:** 2015-05-19

**Authors:** Goo Hyun Baek

**Affiliations:** 1 Department of Orthopaedic Surgery, Seoul National University Hospital, Seoul, 110-744, Korea

## 

Angular deformity of the thumb is most often caused by a delta phalanx or a longitudinally bracheted diaphysis in a triphalangeal thumb. In some angled thumb, however, triangular-shaped secondary ossification center can cause the deformity, which can be called “abnormal triangular epiphysis” of the thumb. A few clinicians have reported this entity, but they do that utilise this term instead classifying class it as a subgroup of delta bone within a triphalangeal thumb.

## Classification (Figure 1)

**Figure 1:**
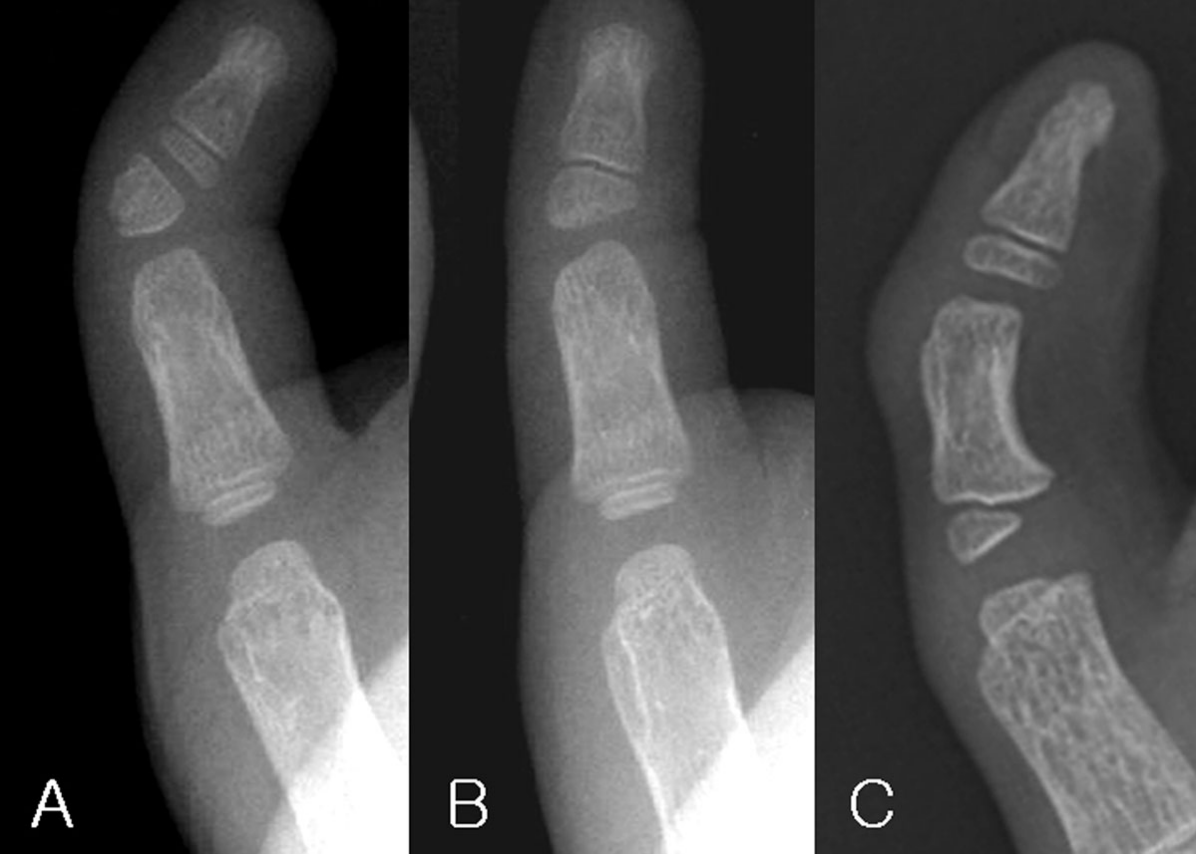
Three types of abnormal ossification centers causing an angulated thumb deformity. (A) Type 1 (extra ossicle), (B) Type 2 (abnormal distal phalanx), and (C) Type 3 (abnormal proximal phalanx).

The classification of the types of angulatory deformity and the variants in ossification of the phalanges of the angled thumbs has not been presented concisely in the literature. Three types of angled thumb from abnormal ossification can be suggested as follows (Figure 1):

Type 1. Distal phalanx normal; proximal phalanx normal; location of abnormal ossification center – separate ossicle.

Type 2. Distal phalanx abnormal; proximal phalanx normal; location of abnormal ossification center – distal phalanx.

Type 3. Distal phalanx normal; proximal phalanx abnormal; location of abnormal ossification center – proximal phalanx.

There are differences between delta phalanx within a triphalangeal thumb (Type 1) and abnormal triangular epiphysis (Type 2), though their early radiographs show similar findings. In a triphalangeal thumb, motion exists between abnormal middle phalanx and distal phalanx, while there is no motion between abnormal triangular epiphysis and distal phalanx because they are one bone. Radiologic distance between abnormal middle phalanx and distal phalanx is also greater than that between abnormal triangular epiphysis and distal phalanx, because the former is a real joint which includes two articular cartilage layers. Finally, the delta phalanx can be clearly distinguishable from a secondary ossification center of the distal phalanx, when well-defined phalangeal epiphyses are clearly evident after a child is 24 to 30 months of age.

## Surgical treatment

For the type 1 angled thumb, simple excision of extra ossicle and reconstruction of collateral ligament is indicated in young children less than 6 years of age. In older children, remodeling power of the joint is limited. Thus resection-arthrodesis is indicated instead of simple excision.

For the type 2 angled thumb, closed wedge corrective osteotomy was done through the epiphysis (intraepiphyseal osteotomy). Proximal phalangeal osteotomy to correct type 2 deformity usually yields a mild serpentine deformity with the IP joint extension and an angular deformity persisted when the IP joint was flexed. As the osteotomy was performed in the proximal phalanx to correct the angular deformity, the axis of joint motion was not perpendicular to the axis of shaft of the phalanx. Therefore, angular deformity persisted with the IP joint flexion and a serpentine deformity occurred. If the osteotomy is performed through the epiphysis, angular deformity can be corrected in just the right place from which the deformity originates, and the axis of the IP joint motion becomes perpendicular to the axis of thumb.

For the type 3 angled thumb, corrective wedge osteotomy of proximal phalanx is indicated. However secondary corrective osteotomy is sometimes necessary due to recurrence of the angular deformity in later age.

## Summary

In type 1 separate ossicle, variable surgical procedures for the delta phalanx can be applied, however, excision and tightening of collateral ligament is recommended in most cases.

In type 2 and 3 abnormal ossification centers, the treatment can depend on the age of the patient. If the angular deformity is seen before the age of one year and the abnormal ossification center is not clearly visible, close observation and periodic follow-up is reasonable. When the abnormal epiphysis is confirmed on radiography, usually between the age of 1 and 2 years, either partial excision of the epiphysis or intra-epiphyseal osteotomy can be done. When the patient is seen late and the remodeling of the joint is not likely to occur, intraepiphyseal osteotomy is recommended with careful attention to the physis and articular cartilage, because it addresses the anatomic deformity and produce better correction regardless of the IP position.
